# Complete Genome Sequence and Methylome of the Type Strain of Shewanella algae

**DOI:** 10.1128/MRA.00559-21

**Published:** 2021-08-05

**Authors:** Christian Tellgren-Roth, Kaisa Thorell, Michael Y. Galperin, Tino Krell, Ute Römling, Åsa Sjöling, Alberto J. Martín-Rodríguez

**Affiliations:** a Department of Immunology, Genetics, and Pathology, Uppsala Genome Center, Uppsala University, Uppsala, Sweden; b Institute of Biomedicine, Department of Microbiology and Immunology, University of Gothenburg, Gothenburg, Sweden; c National Center for Biotechnology Information, National Library of Medicine, National Institutes of Health, Bethesda, Maryland, USA; d Department of Environmental Protection, Estación Experimental del Zaidín, Spanish National Research Council, Granada, Spain; e Department of Microbiology, Tumor and Cell Biology, Karolinska Institutet, Stockholm, Sweden; University of Arizona

## Abstract

We report the complete genome sequence and base modification analysis of the Shewanella algae type strain CECT 5071 (= OK-1 = ATCC 51192 = DSM 9167 = IAM 14159). The genome is composed of a single chromosome of 4,924,764 bp, with a GC content of 53.10%.

## ANNOUNCEMENT

The gammaproteobacterium Shewanella algae was first described as a tetrodotoxin-producing epiphyte of the red alga *Jania* sp. (1), and the type strain was designated OK-1 following the original nomenclature by Kotaki et al. (2). S. algae is the most relevant human pathogen within the genus *Shewanella*, causing bacteremia, otitis, skin and soft tissue infections, and a variety of other diseases, with the emergence of multidrug-resistant isolates being a concern (3, 4). S. algae is also known for its ability to produce secondary metabolites like iron-scavenging siderophores (5). Its versatile physiology is considered a hallmark of the genus *Shewanella*. In the past 3 years, genomes of several clinical and environmental isolates of S. algae have been sequenced (6–9), but no complete genome sequence of a reference strain has been available. We have investigated S. algae from different angles (10–14). Here, we report the complete genome sequence of the S. algae type strain OK-1 (1), which was obtained from the Spanish Type Culture Collection (strain CECT 5071^T^).

The strain was grown in LB medium at 37°C to exponential phase, and the DNA was isolated with the Genomic-tip 500/G kit (Qiagen). Multiplexed sequencing libraries with a target insert size of 8 kb were prepared using the SMRTbell Express template preparation kit v2.0 and barcoded overhang adapter kit 8A (Pacific Biosciences [PacBio], Menlo Park, CA, USA). After removal of adapter dimers, the libraries were sequenced on a Sequel single-molecule real-time (SMRT) cell following the manufacturer’s recommendations (PacBio). Barcode splitting was done using SMRT Link v8.0 (PacBio) and resulted in 622,115 reads totaling 3,045,548,325 bases, with an *N*_50_ value of 7,709 bases. A total of 693,216,159 bases from unique reads were used in the microbial assembly pipeline included in SMRT Link v8.0 (PacBio) with default parameters.

**FIG 1:**
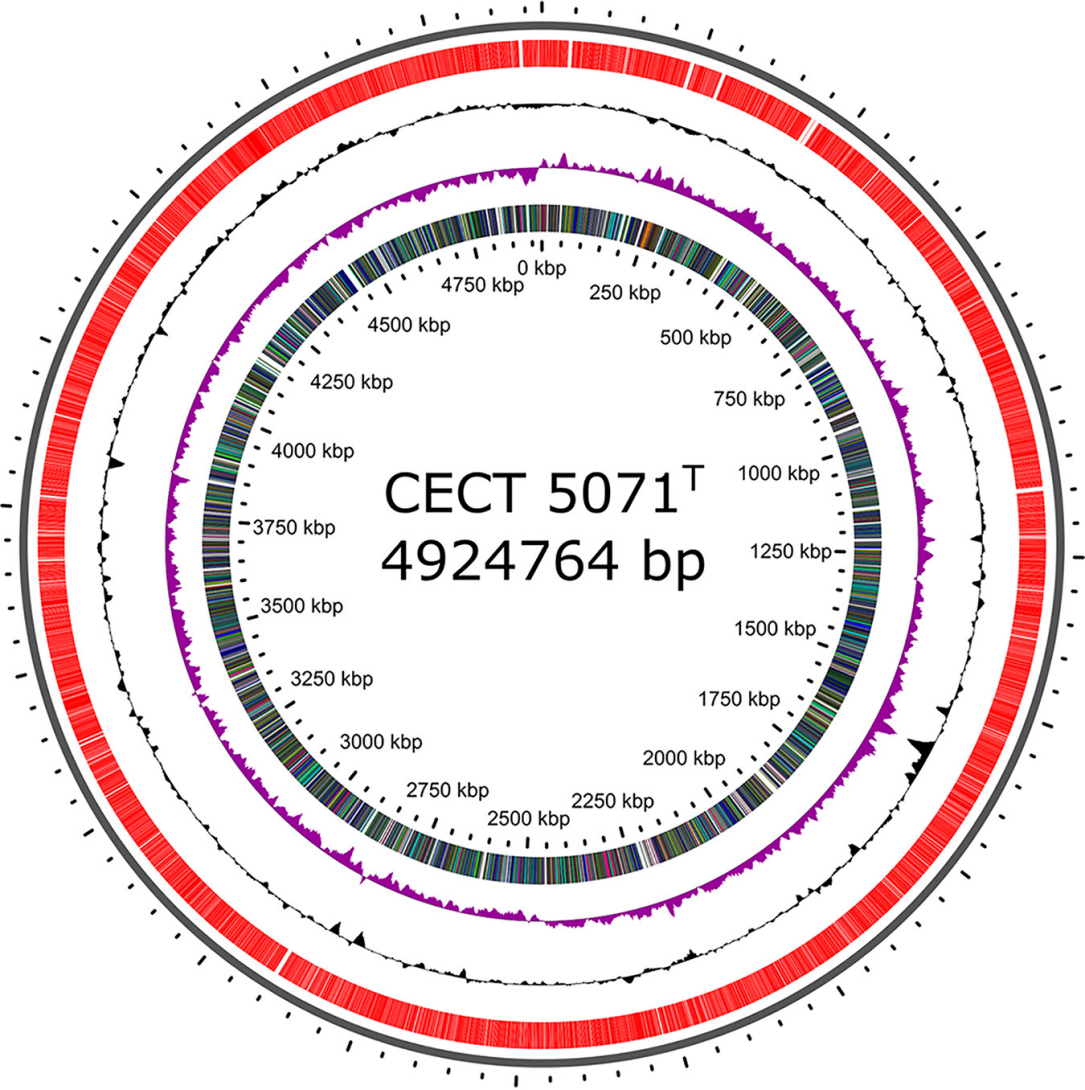
Circular map of the Shewanella algae CECT 5071^T^ chromosome. Circles from outside to inside represent the coding sequences (red), GC content (black), GC skew (purple), and color-coded Clusters of Orthologous Groups (COG) assignments of protein-coding genes. The map was generated with CGView (20).

The genome of the S. algae type strain CECT 5071 is composed of a single chromosome of 4,924,764 bp, with a GC content of 53.10% (Fig. 1). No extrachromosomal elements were detected. Genome annotation by the NCBI Prokaryotic Genome Annotation Pipeline (PGAP) v5.0 (15) predicted 4,400 genes, of which 4,225 are protein-coding genes. A total of 136 RNAs were predicted, including 107 tRNAs, 25 rRNAs (5S, 9 copies; 16S, 8 copies; 23S, 8 copies), and 4 noncoding RNAs. SMRT sequencing offers the possibility of assessing genome-wide DNA methylation patterns. Detected methylation motifs are summarized in Table 1, and the methylome is available in the REBASE database (16).

**TABLE 1:** Summary of methylation motifs detected in the complete genome sequence of S. algae CECT 5071^T^ and associated methyltransferases

Motif	Center position	Modification type	No. detected	No. in genome	Type	Methyltransferase locus tag
GATC	2	m6A	41,451	41,456	II	—^*a*^
TGANNNNNNNTTCC	3	m6A	827	827	Iγ	E1N14_007370
TGGCCA	4	m4C	4,218	8,820	IIα	E1N14_009720

a —, GATC could not be matched unambiguously by REBASE because there is more than one candidate.

Of note, the *rpoS* gene is truncated in the type strain of S. algae (10), consistent with evidence from the draft genomes of equivalent strains, namely, ATCC 51192 (GenBank accession no. GCA_012396675.1), NBRC 103173 (GenBank accession no. GCA_001598875.1), and JCM 21037 (GenBank accession no. GCA_000615045.1), suggesting that this truncation was already present in the original OK-1 isolate. In Escherichia coli, the RpoS protein is the stress sigma factor of the RNA polymerase required for stationary-phase transcription (17). However, natural E. coli *rpoS* mutants exist (18, 19). The ecological and physiological significance of the *rpoS* truncation in the S. algae type strain remains to be determined.

### Data availability.

The complete genome sequence of S. algae CECT 5071^T^ was deposited in DDBJ/ENA/GenBank under the accession no. CP068230. Sequencing raw data are available at the SRA under the accession no. SRR14739658. The methylome of S. algae CECT 5071^T^ is available at the REBASE database under the organism accession no. 46337.
